# Overcoming Target Drift: Development and Validation of a One-Step TaqMan qPCR Assay for Epidemiological Surveillance of *Carpione rhabdovirus* Circulating in Southern China

**DOI:** 10.3390/microorganisms14010126

**Published:** 2026-01-07

**Authors:** Yucong Huang, Zhiyuan Huang, Haoyu Wang, Xiaojuan Li, Xin Liu, Huajian Lin, Zhi Zhang, Xiaofeng Chen, Jichang Jian, Heng Sun

**Affiliations:** 1 Guangdong Provincial Key Laboratory of Aquatic Animal Disease Control and Healthy Culture & Key Laboratory of Control for Diseases of Aquatic Economic Animals of Guangdong Higher Education Institutes, Fisheries College of Guangdong Ocean University, Zhanjiang 524088, China; 2 Guangdong Provincial Animal Disease Prevention and Control Center, Guangzhou 510230, China

**Keywords:** *Carpione rhabdovirus*, differential diagnosis, aquatic viral surveillance, *Trachinotus anak*

## Abstract

*Carpione rhabdovirus* (CAPRV) is an emerging virus within the family *Rhabdoviridae*, posing potential threats to aquaculture species such as golden pompano (*Trachinotus anak*). However, since the 21st century, and for CAPRV strains isolated from marine fish, only a single CAPRV2023 sequence has previously been available in public databases, with no additional sequences reported. Because the virus undergoes genetic variation, relying on this single sequence likely introduced mismatches or off-target risks in earlier detection assay designs. Notably, the previously developed two-step N-targeting detection assay was designed based solely on that single CAPRV2023 sequence. Consequently, this study involved determining and analyzing the N gene sequences from CAPRV isolates gathered from 2023 to 2025, with the aim of pinpointing conserved regions for assay development, and sequence comparisons subsequently verified the existence of mismatches in the primer–probe binding sites of the previous assay. Since quantitative assays in aquatic virology often define copy numbers utilizing either plasmid DNA templates or RNA templates produced via in vitro transcription, which may lead to variations in amplification kinetics and sensitivity, this study compared both standards to ensure reliable quantification across different nucleic acid types. Based on these findings, a one-step TaqMan quantitative PCR (qPCR) assay was developed and validated using dual nucleic acid standards, namely plasmid DNA and in vitro–transcribed RNA. Compared with conventional two-step qPCR, the one-step format combines cDNA synthesis and subsequent DNA amplification in a single sealed tube, thereby effectively preventing cross-contamination, simplifying the workflow, and improving detection efficiency. The assay exhibited strong linearity (R^2^ > 0.99) and consistent amplification efficiencies between 90% and 110%, demonstrating excellent quantitative performance. The detection limits were 2 copies per reaction for plasmid DNA and 20 copies for in vitro–transcribed RNA templates. No cross-reactivity was observed with other aquatic pathogens, and the assay showed strong repeatability and reproducibility (coefficients of variation below 2.0%), providing a sensitive and reliable tool for epidemiological surveillance and the analysis of CAPRV distribution in marine aquaculture systems of southern China.

## 1. Introduction

Golden pompano (*Trachinotus anak*) is a commercially important marine species widely farmed in southern China and Southeast Asia, renowned for its rapid growth, high feed conversion efficiency, and premium flesh quality. It is vital to the aquaculture industry of China, with an annual production projected to exceed 300,000 tons by 2025, according to the Fishery Statistical Yearbook of China (2025) [1]. However, as farming practices intensify, the susceptibility of golden pompano to viral diseases has increased, particularly those caused by rhabdoviruses, leading to significant concerns within the industry [2].

*Rhabdoviridae* represents a large and diverse group of viruses, many of which show broad host ranges spanning both vertebrates and invertebrates [3,4,5,6]. These viruses are known to cause severe diseases in various aquaculture species. For instance, the viral hemorrhagic septicemia (VHS) and spring viremia of carp virus (SVCV) are well-known pathogens that have caused substantial outbreaks in fish populations, leading to high mortality rates and significant economic losses [7,8]. These viruses can not only result in direct fish stock mortality but also incur extensive costs for the management and prevention of outbreaks.

Among the rhabdoviruses, *Carpione rhabdovirus* (CAPRV) is an emerging pathogen that poses a significant threat to golden pompano and other aquaculture species. The first CAPRV strain isolated from marine fish in the 21st century, CAPRV2023 (GenBank accession No. PP050495), was isolated from farmed golden pompano exhibiting hemorrhagic symptoms, including internal bleeding, edema, and lesions in critical organs such as the liver, spleen, and kidneys [2]. These clinical signs were accompanied by high mortality rates, indicating the virus’s potential to cause severe disease outbreaks, especially in intensive aquaculture environments [2]. Subsequent reports indicate an increasing prevalence of CAPRV in aquaculture sites, further underscoring the urgent need for rapid, sensitive, and accurate diagnostic tools [9].

CAPRV belongs to the *Rhabdoviridae* family, sharing common characteristics with other members of the family. *Rhabdoviruses* possess a negative-sense, single-stranded RNA (–ssRNA) genome, and typically have a bullet- or rod- shaped morphology with an enveloped helical nucleocapsid [10]. The genome size ranges from approximately 11 to 16 kb [4,10]. The rhabdovirus genome encodes five major structural proteins: the nucleoprotein (N), which encapsulates the viral RNA; the phosphoprotein (P), which participates in viral replication; the matrix protein (M), which is involved in virion assembly; the glycoprotein (G), which mediates host–cell entry by binding to cellular receptors; and the large polymerase protein (L), which is responsible for RNA replication and transcription [4]. Notably, members of the genus *Novirhabdovirus*, to which CAPRV is classified, additionally encode a non-structural protein (NV) located between the G and L genes. The NV protein has been shown to play important roles in viral pathogenicity and modulation of host antiviral responses, but it is not a structural component of the virion [11]. Within rhabdoviruses, the N protein is a crucial structural component that drives viral replication and assembly; it does this by encapsulating the viral RNA, thereby creating the ribonucleoprotein complex that serves as the necessary template for transcription and replication [12]. In rhabdoviruses, structural proteins are expressed in a defined temporal order during infection, with N being among the earliest and most abundantly expressed proteins, followed by P, M, G, and L, reflecting their respective roles in viral replication, assembly, and host interaction. Compared with other proteins, N is highly expressed and relatively conserved among isolates of the same species, which makes it well-suited for nucleic-acid detection approaches [12,13]. In contrast, although some qPCR assays have targeted the G gene of rhabdoviruses [9,14], this gene is more variable due to immune-selection pressure, which can reduce assay sensitivity and specificity across genetically diverse isolates [15,16]. M and L proteins are expressed at lower levels during infection, further limiting their utility as detection targets [12,13]. Therefore, N is more conserved and highly expressed than other structural proteins, making it a robust target for sensitive and specific CAPRV detection.

However, the RNA virus relies on an RNA-dependent RNA polymerase that lacks proofreading activity, resulting in a relatively high mutation rate and genetic variability among isolates [17]. Sequence variability in the N gene has also been documented in other rhabdoviruses, such as Viral hemorrhagic septicemia virus (VHSV) and Infectious hematopoietic necrosis virus (IHNV). This highlights that although N is the most conserved gene among rhabdoviruses, it still exhibits a certain degree of variation [18,19], and diagnostic assays designed based on a limited number of sequences may experience primer or probe off-targeting due to viral variability, thereby reducing sensitivity and specificity [20]. To date, only the CAPRV2023 genome (No. PP050495), isolated from a marine fish host, sequence has been publicly available [2], and relying solely on this sequence for assay design may lead to off-target amplification or decreased sensitivity when detecting genetically divergent isolates. Notably, our previously developed two-step TaqMan qPCR assay for CAPRV N detection was designed based only on the single publicly available CAPRV2023 sequence [21]. While the N gene is relatively conserved, variations in N sequences among other rhabdoviruses suggested that reliance on a single sequence could introduce mismatches in primer or probe binding sites, potentially affecting assay performance. This concern motivated the sequencing of multiple CAPRV isolates to identify truly conserved regions suitable for robust primer and probe design. To address these issues, CAPRV isolates were collected from naturally infected golden pompanos in different coastal regions of southern China between 2024 and 2025. Ten representative isolates, including the previously preserved CAPRV2023 strain in our laboratory, were subjected to PCR amplification using specific primers and full-length N gene sequence analysis. The N gene of each isolate was amplified and sequenced. Comparative analysis of these sequences allowed the identification of conserved regions suitable for primer and probe design, providing robust targets for molecular detection assays.

Quantitative PCR (qPCR) has become the gold standard for rapid and sensitive detection of viral pathogens in aquaculture due to its high specificity, wide dynamic range, and capacity for accurate quantification [22]. One-step TaqMan qPCR integrates reverse transcription and amplification within a single closed-tube reaction, offering advantages over conventional two-step methods, including fewer handling steps, reduced pipetting errors, lower risk of cross-contamination, and shorter assay times, which enhance efficiency and throughput in diagnostic laboratories [9,23,24].

Accurate viral quantification requires well-characterized nucleic acid standards. Plasmid DNA provides a stable and reproducible template for evaluating amplification consistency, while in vitro–transcribed RNA incorporates the reverse transcription step, more closely reflecting the behavior of viral RNA during actual detection [25]. Comparing both plasmid DNA and RNA standards is therefore critical for comprehensive assay validation. Dual-standard evaluation allows determination of assay sensitivity, efficiency, linearity, and reproducibility across different nucleic acid types, ensuring reliable quantification of viral loads in diverse samples. This approach provides confidence that the developed qPCR assay can reliably detect and quantify genetically diverse CAPRV isolates under practical aquaculture conditions, demonstrating its robustness and suitability for routine surveillance and research applications.

In summary, sequencing multiple CAPRV isolates enabled the identification of conserved N gene regions for robust primer and probe design. The developed one-step TaqMan qPCR provides a rapid, sensitive, and reliable tool for epidemiological surveillance and for analyzing the distribution of CAPRV strains circulating in southern China, thereby supporting early diagnosis and effective disease management in marine aquaculture.

## 2. Materials and Methods

### 2.1. Clinical Samples and Ethics Statement

Tissue collection and processing were performed following the procedures described by Huang et al. [21]. A total of 257 golden pompano specimens, comprising both symptomatic and asymptomatic individuals, were collected from offshore cage-culture facilities in Guangdong (Zhanjiang, Yangjiang) and Guangxi (Beihai, Fangchenggang), China, spanning the period from January 2024 to September 2025. Post-collection, these fish were humanely euthanized using an MS-222 overdose. Subsequently, the spleen, heart, and other designated tissues were harvested under aseptic conditions, instantly preserved by liquid nitrogen freezing, and transported to the laboratory. Portions of each tissue sample were promptly processed for diagnostic assays, with the remaining biological material stored at −80 °C for future analyses. All methodological steps complied with the 2011 NIH Guide for the Care and Use of Laboratory Animals [26] and secured approval from the Animal Ethics Committee of the Guangdong Provincial Key Laboratory of Aquatic Animal Disease Control and Healthy Culture (approval no. 20240105-003; approval date: 5 January 2024).

### 2.2. Total RNA Extraction and cDNA Synthesis

Tissues from the heart and spleen, approximately 50 ± 0.1 mg each, were collected from naturally infected golden pompano (*Trachinotus anak*). Tissue samples were mechanically homogenized in lysis buffer using a MICCRA D-1 homogenizer (Miccra GmbH, Müllheim, Germany) to ensure complete disruption. Total RNA was then extracted from the homogenates using the RNA simple Total RNA Kit (Tiangen, Beijing, China) strictly following the manufacturer’s instructions, without modifications. These kits have been previously validated for RNA extraction and cDNA synthesis from golden pompano tissues in our laboratory.

cDNA synthesis was performed using the FastKing RT Kit (With gDNase) (Tiangen, China) following the manufacturer’s protocol. In the subsequent experiments, to enable a controlled comparison of the sensitivity of the one-step and two-step TaqMan qPCR assays, template input was standardized and the qPCR reaction volume was maintained at 20 µL for both assays. For the one-step assay, 1 µL of RNA was directly added to a 20 µL reaction. For the two-step assay, 5 µL of RNA was reverse-transcribed in a 20 µL reaction, and 4 µL of the resulting cDNA was used as template in a 20 µL qPCR reaction. This approach ensures that the amount of cDNA in the two-step assay is theoretically equivalent to that generated from 1 µL RNA in the one-step assay, allowing a valid comparison of assay sensitivity. It should be noted, however, that actual reverse transcription efficiency may vary slightly, so this equivalence is theoretical.

### 2.3. Design of Primers and Probes for TaqMan qPCR

The full-length N gene of ten CAPRV isolates collected between 2023 and 2025 was amplified using manually designed primers Nke-F and Nke-R, and the PCR products were purified. The primer design was guided by standard principles, including length (18–24 nt), GC content (40–60%), Tm (55–65 °C), avoidance of consecutive nucleotides at the 3′ end [27]. Although the primer Nke-F had slightly lower GC content and the 3′ end of Nke-F contained four G/C nucleotides, the primers successfully amplified the full-length N gene, demonstrating that they were experimentally functional despite minor deviations from ideal parameters. Primer properties, including Tm and potential primer-dimer formation, were manually evaluated based on these guidelines [27]. We note that the forward primer could be further optimized in future designs to improve efficiency, although it performed adequately in the present study.

**Figure 1:**
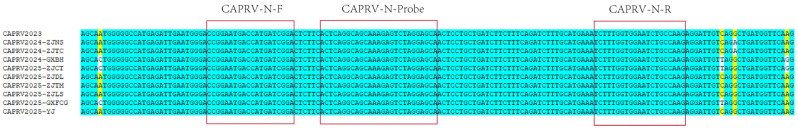
Schematic of primer and probe locations of one-step TaqMan N qPCR. (Blue shading indicates nucleotides conserved across all ten CAPRV isolates. For variable positions, yellow shading indicates nucleotides identical to the reference strain CAPRV2023, while white shading indicates mutated nucleotides relative to CAPRV2023).

**Figure 2:**
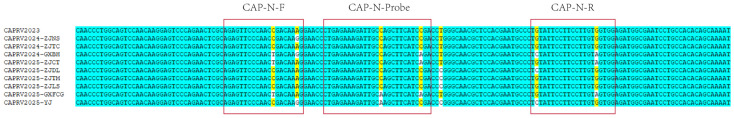
Schematic of primer and probe locations of two-step TaqMan N qPCR. (Blue shading indicates nucleotides conserved across all ten CAPRV isolates. For variable positions, yellow shading indicates nucleotides identical to the reference strain CAPRV2023, while white shading indicates mutated nucleotides relative to CAPRV2023).

The purified PCR amplicons were sequenced using the KB-seq third-generation sequencing platform based on Oxford Nanopore Technology (ONT) at Sangon Biotech (Shanghai, China). All N gene sequences generated in this study have been deposited in the GenBank database. The sequences were aligned and analyzed using DNAMAN (version 5.0, Lynnon Biosoft, San Ramon, CA, USA) to identify conserved regions suitable for assay design. Based on these regions, specific primers and a TaqMan probe were designed using the GenScript online qPCR design tool (https://www.genscript.com.cn/tools/real-time-pcr-taqman-primer-design-tool, accessed on 1 November 2025) with the following parameters: PCR amplicon size 50–200 bp, primer Tm 52–60 °C, and probe Tm 62–70 °C, and potential self-complementarity and primer-dimer formation were evaluated. The resulting qPCR primers, CAPRV-N-F and CAPRV-N-R, together with the TaqMan probe (CAPRV-N-Probe), target a 110 bp amplicon within a conserved central region of the N gene, ensuring specificity and sensitivity for CAPRV detection. BLAST (https://blast.ncbi.nlm.nih.gov/Blast.cgi, accessed on 9 November 2025) analysis against the NCBI nucleotide database was performed for all primers and probes to confirm their specificity and exclude potential cross-reactivity. In addition, the full-length N gene sequences of the ten CAPRV isolates obtained in this study were compared with the primer and probe binding sites of the previously established two-step TaqMan N-gene qPCR assay to identify any nucleotide differences. The TaqMan probe carried a 5′ FAM fluorophore and a 3′ BHQ1 quencher. Detailed sequences of the primers and probe used in this study are provided in Table 1, and the positions of the primers and probes for both the one-step and two-step assays within the CAPRV N gene are illustrated in Figure 1 and Figure 2.

### 2.4. Plasmid Construction for Standard Quantification

To generate a plasmid standard for qPCR, the full-length N gene (1203 bp) of the CAPRV2023 original strain was amplified using primers Nke-F and Nke-R in the PCR reaction. The amplified product was purified with the SanPrep Column PCR Product Purification Kit (Sangon, Shanghai, China) and cloned into the pESI-Blunt Simple vector using the Hieff Clone^®^ Zero TOPO-Blunt Simple Cloning Kit (Yeasen, Shanghai, China). The recombinant plasmid was transformed into DH5α cells, which were subsequently plated on LB agar containing ampicillin for selection. Positive colonies were initially screened by colony PCR, and the correct insertion of the N gene fragment was confirmed by Sanger sequencing. Plasmids from verified positive clones were extracted using the FastPure Plasmid Mini Kit (Vazyme, Nanjing, China), adjusted to a final concentration of 2 × 10^12^ copies/μL, and stored at −80 °C for future use.

### 2.5. Standard RNA Template Preparation

RNA standards representing the negative-sense orientation of the CAPRV N gene were prepared following the general workflow described by Huang et al. [21], with modifications in reagents and kits specific to the current study. Briefly, a 537 bp fragment encompassing the target region was amplified from cDNA using primers, including a T7 promoter on the reverse primer. The PCR product was purified and used as a template for in vitro transcription with a T7 RNA polymerase kit (Vazyme, China). Residual DNA was removed by DNase I treatment, and the resulting RNA transcripts were purified and quantified using the Equalbit RNA BR Assay Kit in combination with a Qubit 4 Fluorometer (Thermo Fisher Scientific, Waltham, MA, USA). The RNA concentration was adjusted to 2 × 10^8^ copies/μL, and aliquots were stored at −80 °C until use.

### 2.6. Development and Optimization of TaqMan qPCR Conditions

Optimization of the TaqMan qPCR assay was performed using total RNA extracted from naturally infected golden pompano, as described in Section 2.2. Reverse transcription and PCR amplification were conducted sequentially in a single tube using the AccurSTART U+ One Step RT-qPCR Probe Kit (FOR FAST) (Vazyme, China). Following the manufacturer’s recommended conditions, cDNA was first synthesized by reverse transcription at 55 °C for 15 min. This was followed by an initial denaturation step at 95 °C for 30 s. Similarly, following the manufacturer’s recommendations, the annealing temperature of 60 °C was used for qPCR amplification.

To determine the optimal probe and primer concentrations, a series of reactions was first performed using different probe concentrations of 50 nM, 100 nM, 150 nM, 200 nM, and 250 nM while maintaining constant primer concentrations of 0.2 μM. After the optimal probe concentration was identified, primer concentrations were further optimized at 0.1 μM, 0.2 μM, 0.4 μM, and 0.6 μM. Each reaction was performed in triplicate using the LightCycler^®^ 96 Real-Time PCR System (Roche, Solna, Sweden). The optimal reaction conditions were defined as those yielding the lowest threshold cycle (Ct) values, relatively higher amplification efficiency, and lower standard deviation (SD). These optimized parameters were subsequently applied to all downstream analyses.

### 2.7. Construction of Standard Curves and Evaluation of qPCR and Conventional PCR Sensitivity and Reproducibility

To ascertain the operational characteristics and lower detection limits of the TaqMan qPCR assay, a series of plasmid DNA standards (2 × 10^8^ to 2 × 10^0^ copies/μL) and in vitro-transcribed RNA standards (2 × 10^8^ to 2 × 10^1^ copies/μL) were generated. Each dilution underwent triplicate evaluation under optimized reaction conditions. Standard curves were formulated by correlating the logarithmic value of the copy number with the average Ct values from each concentration, which allowed for the derivation of the correlation coefficient (R^2^) and the quantification of amplification efficiency. Furthermore, an average amplification curve was created for each concentration by computing the mean fluorescence signals from three technical replicates at every cycle, thereby facilitating the evaluation of amplification uniformity and the establishment of the detection threshold.

Simultaneously, the sensitivity of conventional PCR was investigated utilizing the same plasmid DNA and in vitro-transcribed RNA templates. Initially, RNA standards were reverse-transcribed into cDNA using the HiScript IV 1st Strand cDNA Synthesis Kit (+gDNA wiper) (Vazyme, China) prior to their amplification. PCR assays were executed with the 2× Rapid Taq Plus Master Mix (Dye Plus) (Vazyme, China), following a thermal profile that commenced with an initial denaturation at 95 °C for 2 min, proceeded with 35 cycles of 98 °C for 10 s, 55 °C for 30 s, and 68 °C for 10 s, and concluded with a final extension at 68 °C for 5 min.

The reproducibility of the qPCR assay was assessed by evaluating both intra-assay and inter-assay variability using the plasmid DNA and in vitro-transcribed RNA standards. Intra-assay variability was determined by analyzing three replicates of each dilution within a single experimental session, whereas inter-assay variability was established by conducting three separate runs on different days. The precision and consistency of the assay were quantified by calculating the coefficient of variation (CV%) of the Ct values.

### 2.8. Specificity Evaluation of the One-Step TaqMan qPCR Assay

The specificity of the one-step TaqMan qPCR assay was assessed using nucleic acids from eleven aquatic pathogens commonly found in fish. Total RNA was isolated from *Siniperca chuatsi* rhabdovirus (SCRV), VHSV, Spring viremia of carp virus (SVCV), IHNV, and Nervous necrosis virus (NNV). Viruses such as SCRV, VHSV, SVCV, and IHNV were included because they are phylogenetically related to rhabdoviruses and could potentially produce cross-reactivity in the assay [2]. NNV, a +ssRNA virus, was included because it has been reported in golden pompano aquaculture and represents a relevant environmental pathogen [28].

In addition, genomic DNA was prepared from six bacterial pathogens commonly found in golden pompano farms, including *Streptococcus agalactiae*, *Vibrio alginolyticus*, *Streptococcus iniae*, *Vibrio harveyi*, *Lactococcus garvieae*, and *Vibrio vulnificus* [29,30,31]. Each extracted sample served as the template for amplification in the one-step qPCR system. Spleen and heart tissues from CAPRV-infected golden pompano (*Trachinotus anak*) were used as the positive control, and nuclease-free water served as the negative control. All tests were performed in triplicate to confirm the reproducibility of the assay.

### 2.9. Comparative Analysis of One-Step and Two-Step TaqMan qPCR Using Representative CAPRV Strains

To further evaluate and compare the analytical performance of the newly developed one-step TaqMan qPCR assay targeting the CAPRV N gene with our previously established two-step TaqMan N gene qPCR method [21], ten representative CAPRV isolates collected between 2024 and 2025, including the original reference strain, were selected for testing. These isolates were chosen to reflect the genetic diversity and epidemiological variation observed among circulating CAPRV strains in southern China. For each isolate, RNA templates were analyzed using both assays with three independent technical replicates per method. The resulting Ct values were used to assess differences in detection sensitivity, quantification consistency, amplification efficiency, and overall diagnostic performance. This comparative analysis provided a comprehensive evaluation of whether the one-step assay offers improved sensitivity, robustness, and operational efficiency compared with the two-step approach.

### 2.10. Epidemiological Sampling and Molecular Detection of CAPRV in Golden Pompanos

From January 2024 to September 2025, a total of 257 golden pompanos (*Trachinotus anak*) specimens, including moribund, diseased, and apparently healthy individuals from offshore cage farms, were collected in Guangdong (Zhanjiang, Yangjiang) and Guangxi (Beihai, Fangchenggang) provinces, China. Samples were classified as diseased or moribund based on the observation of clinical signs such as hemorrhage, exophthalmia, skin ulceration, pale gills, and abnormal swimming behavior. In contrast, healthy fish showed no visible lesions or behavioral abnormalities. Both conventional PCR and the newly developed one-step TaqMan qPCR assay established in this study, as well as our previously developed two-step TaqMan qPCR assay targeting the N gene [21], and the previously reported one-step TaqMan qPCR method by Sun et al., which targets the G gene [9], were employed to detect CAPRV in the collected samples following the procedures described in Section 2.2. The obtained results were used to compare the detection sensitivity and diagnostic performance of the different assays and to investigate the incidence and prevalence of CAPRV infection among golden pompano populations in the coastal regions of southern China over the past two years.

### 2.11. Statistical Analysis

All data were presented as mean ± standard deviation. Statistical analyses were performed using SPSS 27 (IBM Corp., Armonk, NY, USA). One-way analysis of variance was used to compare Ct values from the primer concentration optimization experiments, and Ct values from ten viral strains were analyzed using the same procedure. Tukey’s post hoc test was applied for multiple comparisons. Statistical significance was defined at *p* < 0.05, and differences among groups were indicated with different letters.

## 3. Results

### 3.1. Conservation of Primer and Probe Binding Regions Among CAPRV Strains from Different Regions

The N gene fragments of ten representative CAPRV strains, collected from January 2024 to September 2025 from different epidemic regions—including Zhanjiang (Liusha Port, Tongming Port, Caotan Town, Dongli Town, Techeng Island, and Nansan Island) and Yangjiang in Guangdong Province, as well as Beihai and Fangchenggang in Guangxi Province—were aligned to assess sequence conservation within the primer and probe binding sites. All sequences have been deposited in GenBank under accession numbers PX766083–PX766091. The alignment showed that the target region of the one-step TaqMan N-gene qPCR assay developed in this study was highly conserved among all strains, with no nucleotide variation observed in the primer or probe binding regions (Figure 1). The binding positions of the primers and probe are indicated by boxes in the alignment.

In addition, the sequences were compared with the primer and probe binding sites of our previously established two-step TaqMan N-gene qPCR assay [21]. This analysis revealed that six isolates (2024-ZJNS: PX766091; 2024-GXBH: PX766083; 2025-ZJCT: PX766087; 2025-ZJDL: PX766086; 2025-GXFCG: PX766089; 2025-YJ: PX766088) carried nucleotide mutations within the two-step assay’s primer or probe binding regions, whereas the remaining isolates (2024-ZJTC: PX766090; 2024-ZJTM: PX766084; 2025-ZJLS: PX766085) had no such mutations (Figure 2). These results indicate that the primers and probe of the one-step assay exhibit strong sequence universality for detecting CAPRV strains circulating in southern China, while the two-step assay may be affected by sequence variations in some viral strains.

### 3.2. Optimization of Primer and Probe Concentrations

**Figure 3:**
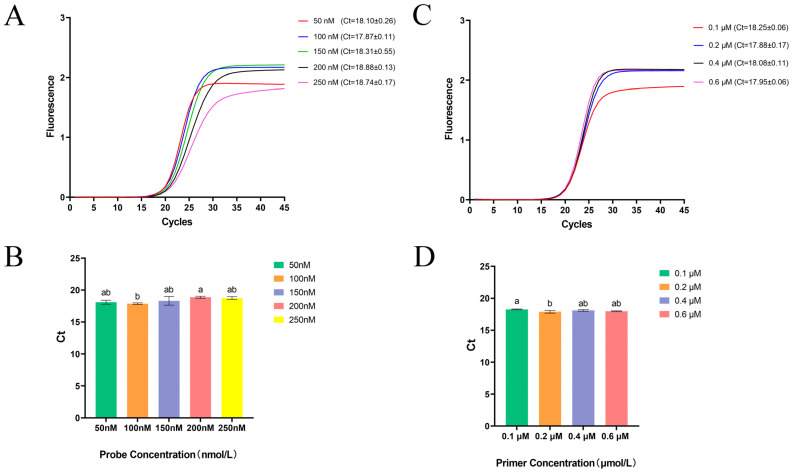
(**A**) Amplification curves for probe concentration optimization. (**B**) Histogram showing probe concentration optimization; different lowercase letters above bars indicate significant differences (*p* < 0.05) among three replicates under the same condition. (**C**) Amplification curves for primer concentration optimization. (**D**) Histogram showing primer concentration optimization; different lowercase letters above bars indicate significant differences (*p* < 0.05) among three replicates under the same condition.

The concentrations of the probe and primers were systematically optimized while maintaining the annealing temperature at the recommended 60 °C. First, with primer concentrations fixed at 0.2 μM, probe concentrations of 50, 100, 150, 200, and 250 nM were tested, and 100 nM was selected as optimal based on the lowest Ct values. Next, with the probe fixed at 100 nM, primer concentrations of 0.1, 0.2, 0.4, and 0.6 μM were evaluated, and 0.2 μM, which produced the lowest Ct, was selected as the final working concentration. The optimized amplification program consisted of reverse transcription at 55 °C for 15 min, initial denaturation at 95 °C for 30 s, followed by 45 cycles of PCR, each including denaturation at 95 °C for 10 s and annealing/extension at 60 °C for 30 s with fluorescence signal acquisition. These optimized reaction conditions were subsequently applied to all downstream experiments (Figure 3).

### 3.3. Calibration Standard Curve of the TaqMan qPCR Assay

**Figure 4:**
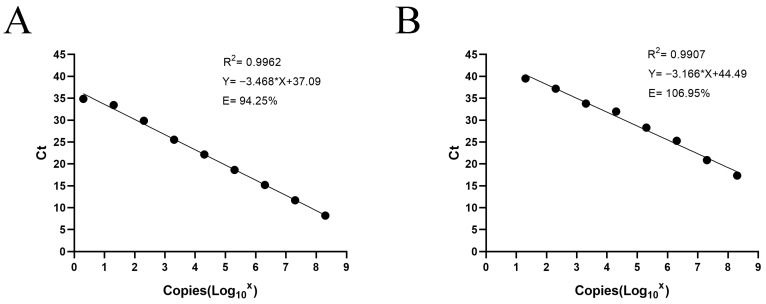
Standard curves of the TaqMan qPCR assay for CAPRV detection. (**A**) Standard curve based on serially diluted plasmid DNA; (**B**) Standard curve based on serially diluted in vitro-transcribed RNA.

Standard curves for the developed TaqMan qPCR assay were constructed using plasmid DNA (2 × 10^8^ to 2 × 10^0^ copies/μL) and in vitro-transcribed RNA (2 × 10^8^ to 2 × 10^1^ copies/μL) standards to evaluate the quantitative performance of the method. Each dilution was tested in triplicate, and mean Ct values were used to plot the standard curves. For plasmid DNA, the regression equation was Y = −3.468X + 37.09, with R^2^ = 0.9962 and an amplification efficiency of 94.25%. For in vitro-transcribed RNA, the regression equation was Y = −3.166X + 44.49, with R^2^ = 0.9907 and an amplification efficiency of 106.95%. Both curves displayed strong linearity and reproducibility, confirming the high accuracy and quantitative reliability of the developed TaqMan qPCR assay for CAPRV detection (Figure 4). A generally linear relationship was observed across the RNA transcript dilution series. A slight deviation from linearity was noted at the two highest RNA concentrations, which were located at the upper limit of the assay’s linear dynamic range. Overall, the assay exhibited acceptable correlation coefficients and amplification efficiencies within the defined quantitative range.

### 3.4. Evaluation of the TaqMan Quantitative Polymerase Chain Reaction (qPCR)

Standard curves and amplification curves were generated using plasmid DNA and in vitro-transcribed RNA templates to evaluate the performance of the optimized TaqMan qPCR assay. Plasmid DNA was diluted from 2 × 10^8^ to 2 × 10^0^ copies/μL, and in vitro-transcribed RNA was diluted from 2 × 10^8^ to 2 × 10^1^ copies/μL. Each dilution was tested in triplicate.

**Figure 5:**
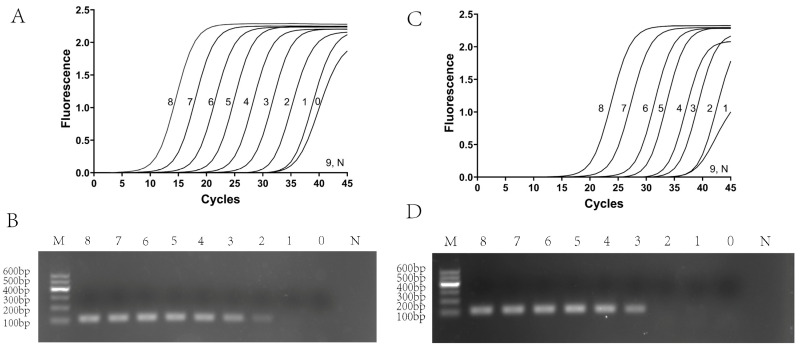
Sensitivity analysis of the TaqMan qPCR assay and the conventional PCR. (**A**) TaqMan qPCR amplification curves based on plasmid DNA standards. 0–8: 2 × 10^0^ to 2 × 10^8^ copies/μL; 9: negative control; (**B**) Conventional PCR amplification based on plasmid DNA standards. Lane 0–8: 2 × 10^0^ copies/μL to 2 × 10^8^ copies/μL; Lane M: DL 600 bp DNA marker; N: negative control. (**C**) TaqMan qPCR amplification curves based on in vitro-transcribed RNA standards. 1–8: 2 × 10^1^ to 2 × 10^8^ copies/μL; 9: negative control. (**D**) Conventional PCR amplification based on in vitro-transcribed RNA standards. Lanes 0–8: 2 × 10^0^ to 2 × 10^8^ copies/μL; M: DL 600 bp DNA marker; N: negative control.

Amplification curves were obtained by averaging the fluorescence signals at each cycle. The detection limit of the TaqMan qPCR was 2 × 10^0^ copies/μL for plasmid DNA and 2 × 10^1^ copies/μL for in vitro-transcribed RNA. In contrast, conventional PCR achieved detection limits of 2 × 10^2^ copies/μL for plasmid DNA and 2 × 10^3^ copies/μL for in vitro-transcribed RNA. This indicates that the assay improved sensitivity approximately 100-fold over conventional PCR (Figure 5). Although the sensitivity of in vitro-transcribed RNA is inferior to that of plasmid DNA, it reflects the reverse transcription step and thus more closely represents actual viral detection, while still providing robust sensitivity. Reproducibility was assessed for both templates.

For plasmid DNA, intra-assay standard deviations (SDs) ranged from 0.04 to 0.56, with coefficients of variation (CVs) of 0.16–1.66%. Inter-assay SDs ranged from 0.09 to 0.52, with CVs of 0.58–1.56%. For in vitro-transcribed RNA, intra-assay SDs ranged from 0.12 to 0.66, with CVs of 0.19–1.94%. Inter-assay SDs ranged from 0.12 to 0.72, with CVs of 0.63–1.67% (Table 2 and Table 3). These results demonstrate that the TaqMan quantitative polymerase chain reaction (qPCR) assay is highly reproducible and repeatable for both plasmid and RNA templates.

### 3.5. Specificity of the One-Step TaqMan qPCR

The specificity of the one-step TaqMan qPCR assay was evaluated using genomic RNA or DNA extracted from eleven common aquatic pathogens. Under the optimized reaction conditions, clear amplification signals were observed exclusively for CAPRV, whereas no detectable amplification occurred for any of the other tested pathogens. These results demonstrate that the assay specifically detects CAPRV without cross-reactivity, confirming its high analytical specificity.

### 3.6. Comparative Analysis of One-Step and Two-Step TaqMan N-Gene qPCR Methods Using Diverse CAPRV Isolates

**Figure 6:**
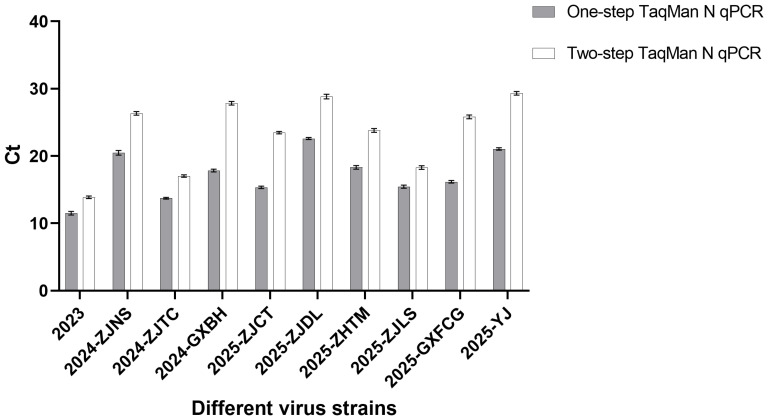
Comparison of Ct Values for Ten CAPRV Isolates Using One-Step and Two-Step TaqMan N qPCR Assays.

To assess the performance differences between the newly developed one-step TaqMan qPCR assay and our previously established two-step TaqMan N-gene qPCR method [21], ten representative CAPRV isolates collected from 2024 to 2025—including the original reference strain CAPRV2023—were selected. For all ten isolates, the one-step assay consistently outperformed the two-step method, producing lower Ct values across three independent technical replicates, indicating higher sensitivity and more efficient amplification (Figure 6). Notably, isolates (2024-ZJNS, 2024-GXBH, 2025-ZJCT, 2025-ZJDL, 2025-GXFCG, and 2025-YJ) in which the primer or probe binding sites of the previously established two-step TaqMan N-gene qPCR carried nucleotide mutations exhibited larger Ct differences between the one-step and two-step assays compared with isolates (2024-ZJTC, 2024-ZJTM, 2025-ZJLS) without such mutations (Table 4). These results demonstrate that the one-step assay exhibits higher sensitivity compared with the two-step method, and that the sensitivity of the two-step assay is further reduced when sequence variations occur in the primer/probe binding regions, highlighting the overall superior analytical performance of the one-step method developed in this study.

### 3.7. Application of the One-Step TaqMan qPCR and Epidemiological Investigation

**Figure 7:**
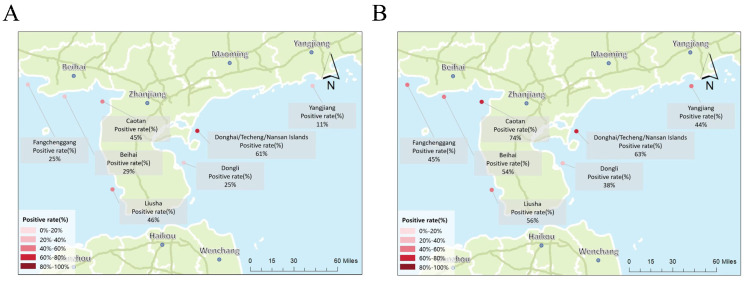
Epidemiological survey of CAPRV in different regions of South China. (**A**) Positive detection rates in Guangdong and Guangxi during 2024. (**B**) Positive detection rates in Guangdong and Guangxi during 2025.

A total of 257 golden pompano samples were collected from offshore cage farms in Guangdong Province (Zhanjiang: Caotan/Liusha/Dongli and Donghai/Nansan/Techeng Island, Yangjiang, Jiangmen) and Guangxi Province (Beihai, Fangchenggang) from January 2024 to September 2025. Spleen and heart tissues were tested using the newly developed one-step TaqMan qPCR assay and conventional PCR, as well as a previously reported one-step TaqMan qPCR assay targeting the G gene and a prior two-step TaqMan qPCR assay targeting the N gene. The epidemiological results shown in the map (Figure 7) represent the positive detection rates based on the one-step TaqMan qPCR assay established in this study, which targets the N gene of CAPRV.

For samples collected in 2024, the overall positive detection rates of CAPRV were 52.63% (40/76) in Zhanjiang and 11.11% (1/9) in Yangjiang, Guangdong Province, and 27.27% (2/7) in Beihai and 25.00% (1/4) in Fangchenggang, Guangxi Province. From January to September 2025, positive rates increased in most regions: 61.98% (75/121) in Zhanjiang and 43.75% (7/16) in Yangjiang, Guangdong Province, and 53.84% (7/13) in Beihai and 45.45% (5/11) in Fangchenggang, Guangxi Province, indicating an upward trend in CAPRV prevalence in southern China (Figure 7).

**Figure 8:**
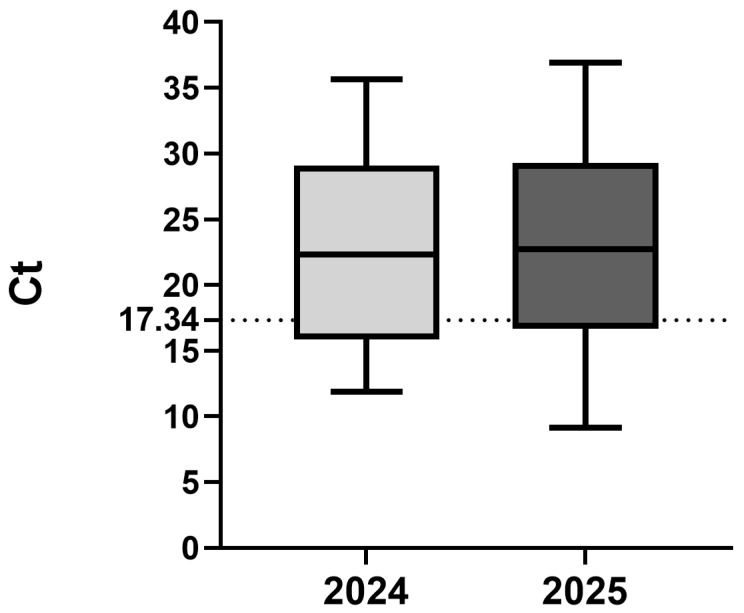
Distribution of Ct values obtained from One-Step TaqMan N qPCR CAPRV-positive field samples collected in 2024 (n = 44) and 2025 (n = 94). The dashed line indicates the upper limit of the validated linear dynamic range defined using RNA transcript standards. Ct values below this limit represent samples with high viral burdens and were interpreted qualitatively.

To further evaluate assay performance under field conditions, Ct values of all positive samples detected by the newly developed one-step TaqMan qPCR assay were summarized. Positive field samples collected in 2024 (n = 44) and 2025 (n = 94) showed a broad distribution of Ct values (Figure 8). The linear dynamic range of the assay was defined using RNA transcript standards and validated up to 2 × 10^8^ copies per reaction (Ct = 17.34). Most field-positive samples fell within this validated range, while a subset exhibited Ct values below the upper limit, reflecting high viral burdens. These samples were therefore interpreted qualitatively as strongly positive rather than being subjected to precise quantitative estimation.

Over the two-year period, the overall positive detection rate using the N gene–targeting one-step TaqMan qPCR assay developed in this study was 53.70% (138/257), whereas the previously reported one-step TaqMan G qPCR method by Sun et al. [9], which targets the G gene, detected 45.14% (116/257) of samples and two-step TaqMan N qPCR method by Huang et al. [21], detected 48.25% (124/257) of samples. In contrast, conventional PCR detected only 34.63% (89/257) of samples (Table 5). These findings demonstrate that the newly developed N gene–based TaqMan qPCR assay achieved the highest detection rate among the three methods, exhibiting greater analytical sensitivity and reliability for CAPRV surveillance in golden pompano populations.

## 4. Discussion

In this study, the one-step TaqMan qPCR assay developed in this study represents a significant methodological advance for the detection of *Carpione rhabdovirus* in golden pompano. CAPRV2023, first identified in southern China in 2023, is a newly emerging rhabdovirus associated with hemorrhagic symptoms, tissue necrosis, and high mortality in aquaculture populations, resulting in severe economic losses across the region [2]. Since its discovery, epidemiological evidence has indicated that the pathogen is spreading rapidly among offshore cage farms in the southern marine regions of China, suggesting its adaptation to marine aquaculture environments and potential for regional persistence [9]. At present, no effective vaccines or antiviral strategies have been developed, and disease control primarily relies on husbandry management and biosecurity measures. Therefore, establishing a sensitive and specific molecular diagnostic assay capable of detecting CAPRV during the early stages of infection is of critical importance for timely intervention and sustainable development of the golden pompano industry. In this context, a robust molecular tool that can be readily applied to routine field screening and epidemiological investigations is essential for understanding CAPRV circulation and transmission dynamics in aquaculture systems.

Although SYBR Green and TaqMan qPCR assays targeting the G gene of CAPRV have been previously reported [2,9], the frequent antigenic variation in this gene may reduce detection sensitivity or even cause off-target effects when applied to genetically variable field strains. In other rhabdoviruses, it has been reported that mutations in the G gene can lead to inconsistent qPCR performance among different viral strains, thereby rendering N gene–targeted assays generally more reliable than those targeting G and achieving higher positive detection rates [32]. A similar pattern was also observed in this study, where epidemiological testing of CAPRV using N gene– and G gene–targeted assays confirmed that the N gene–based method consistently produced higher positive detection rates. Given its evolutionary stability, strong sequence conservation, and the highest transcriptional abundance among rhabdovirus genes, the N gene represents the most suitable target for the development of a robust and reliable CAPRV detection assay intended for large-scale surveillance and long-term monitoring of circulating viral strains.

As only the genome sequence of the prototype CAPRV2023 strain—the first CAPRV isolate identified from marine fish (golden pompano) in the 21st century—was available when the virus was initially reported, early detection assays were necessarily designed based on this single reference sequence [2]. However, since RNA viruses rely on RNA-dependent RNA polymerases that lack proofreading activity, they are highly prone to genetic variation [33]. Mutations occurring within primer or probe binding regions may introduce potential diagnostic bias, thereby reducing assay sensitivity or causing false-negative results [34]. As a negative-sense RNA virus, CAPRV is expected to undergo frequent nucleotide substitutions over time, even within highly conserved genomic regions such as the N gene, a phenomenon that was confirmed in this study. Similar cases of N gene variation have been documented in other aquatic *rhabdoviruses* [35,36]. To address this limitation, we sequenced the N gene from ten representative CAPRV isolates collected from different coastal regions of Guangdong and Guangxi provinces and performed multiple-sequence alignment to identify highly conserved motifs. Primers and a TaqMan probe were then designed within these conserved regions to ensure broad detection coverage across circulating genotypes. This approach substantially improved assay inclusivity and robustness, representing the first CAPRV detection system developed using multiple field isolates rather than a single reference sequence—a critical advancement toward greater diagnostic reliability and long-term applicability in epidemiological surveillance programs.

Notably, CAPRV-related viruses have been reported from distinct host species and geographic regions over a long temporal span [2]. To date, only one full-length CAPRV genome from a non-marine host has been publicly deposited in GenBank, which was isolated from the freshwater salmonid *Salmo carpione* in Italy in 1988 and represents a historically, geographically, ecologically (freshwater), and host-distinct lineage [2]. Being temporally, ecologically, and host-distinct, this historical Italian isolate is considered different from contemporary marine CAPRV strains, which likely reflects long-term geographic separation, host-specific adaptation, and the rapid evolutionary dynamics characteristic of negative-sense RNA viruses. Consequently, this Italian isolate was not included in the design of the present assay, which was specifically optimized for CAPRV strains currently circulating in golden pompano aquaculture systems in southern coastal China. Accordingly, the primers and probe were deliberately designed based on multiple recent CAPRV isolates obtained from golden pompano, thereby maximizing diagnostic sensitivity and reliability for these marine strains. This approach underscores the importance of developing molecular diagnostic assays tailored to contemporary, region- and host-relevant viral populations rather than relying exclusively on genetically distant historical reference strains. Such regionally optimized assay design is particularly important for accurately assessing pathogen distribution and prevalence in marine aquaculture settings. Interestingly, studies have suggested that snakehead rhabdovirus (SHRV) is closely related to CAPRV [2,37], highlighting the potential for broader cross-reactivity studies in the future.

Additionally, our previously developed two-step TaqMan qPCR assay targeting the N gene was designed solely based on the single CAPRV2023 sequence [21]. Comparative analysis of the N gene sequences from ten representative isolates revealed that several strains contained mismatches at the primer and probe binding sites of the previous two-step assay, whereas the conserved regions targeted by the newly developed one-step assay showed no such mismatches. When these isolates were tested using both assays, the one-step method consistently produced lower Ct values across all strains. This observation suggests that the improved detection performance of the one-step assay may be influenced by both the avoidance of primer/probe mismatches in some strains and the different methodological factors. Indeed, studies in other viral systems have shown that one-step RT-qPCR outperforms two-step RT-qPCR, producing significantly lower Ct values [38,39]. In addition, one-step RT-qPCR is more effective at detecting low-abundance targets, with two-step RT-qPCR often resulting in a 3–6 cycle delay in Ct values compared to the one-step method [40]. Furthermore, in strains containing primer/probe mismatches, the Ct difference between the new and old assays was more pronounced, which may reflect the potential impact of viral sequence variation on the reduced detection sensitivity of the previous assay. These findings underscore the importance of designing assays based on multiple isolates and conserved regions to achieve robust and reliable detection across genetically diverse viral populations encountered during field-based epidemiological investigations. Notably, the higher sensitivity of the one-step TaqMan RT-qPCR developed in this study compared with previously reported two-step assays may result from multiple factors, including differences in reaction format (one-step vs. two-step), primer/probe design, or a combination of both. Because the new assay was developed using newly designed primers and probe, whereas the two-step assay used previously published primer/probe sets, it is not possible to determine the relative contribution of reaction format versus primer/probe design to the improved sensitivity. Nevertheless, the current assay provides a practical and robust detection tool for circulating CAPRV strains. Future studies performing systematic, factorial comparisons of primer/probe sets across one-step and two-step formats could clarify the specific factors driving assay performance.

Compared with the conventional two-step qPCR approach, the one-step TaqMan qPCR assay established in this study offers clear advantages in workflow simplicity, analytical precision, and contamination control. In the two-step format, RNA is first reverse transcribed into cDNA in one reaction, followed by separate amplification in another, which not only prolongs the experimental process but also increases the risk of cross-contamination and pipetting errors [9,24]. The one-step system integrates both reverse transcription and amplification within a single sealed tube, effectively reducing hands-on time, minimizing sample loss, and improving biosafety [24]. Additionally, the adoption of TaqMan hydrolysis probe chemistry provides triple-layer specificity—requiring two primers and one fluorescent probe to simultaneously recognize the target sequence [41]. This design eliminates the risk of nonspecific fluorescence signals caused by primer-dimer formation or off-target amplification, which are common in SYBR Green-based assays [42,43,44,45]. Although the reagent cost per reaction for TaqMan qPCR is higher than that of conventional PCR and SYBR Green qPCR—for example, approximately US$1.50 per reaction in this study—its superior sensitivity compared with conventional PCR and lower false-positive rate compared with SYBR Green qPCR make it particularly suitable for high-throughput diagnostic screening and routine epidemiological surveillance programs where accuracy and reliability are of critical importance [42,43,46].

To achieve optimal performance under realistic diagnostic conditions, reaction optimization was performed using RNA extracted from naturally infected golden pompano. This approach better reflects the complex biological matrices encountered in field samples, ensuring that the optimized parameters are applicable to real diagnostic settings. Through systematic evaluation of primer and probe concentrations, 0.2 μM primers and 100 nM TaqMan probe were determined as the optimal working concentrations, which produced the lowest Ct values and the highest amplification efficiency. These results demonstrate the robustness, reproducibility, and practical applicability of the optimized one-step TaqMan qPCR assay for routine field diagnostics.

To comprehensively characterize the assay’s analytical performance, both plasmid DNA and in vitro-transcribed RNA standards were generated to construct dual standard curves. Each standard served a specific validation purpose. Plasmid DNA, being highly stable and precisely quantifiable, is ideal for evaluating amplification efficiency and linearity [47]. However, plasmid standards bypass the reverse transcription step, thus underestimating variability in cDNA synthesis [48]. In contrast, RNA standards simulate the entire diagnostic workflow—from reverse transcription through amplification—providing a biologically realistic estimate of detection sensitivity. The combined use of both standard types offered complementary insights: plasmid curves assessed intrinsic qPCR performance, whereas RNA curves reflected total system sensitivity under diagnostic conditions. In this study, detection limits of 20 copies/μL for plasmid DNA and 200 copies/μL for RNA were achieved, representing approximately two orders of magnitude higher sensitivity than conventional PCR. Both standard curves exhibited excellent linearity (R^2^ > 0.99) and efficiencies within the 90–110% range, confirming the quantitative reliability of the method. The sensitivity of RNA standards is inferior to that of plasmid standards, which is consistent with previous reports on other viral qPCR assays. This is because the reverse transcription step required for RNA introduces additional variability and potential template loss, resulting in higher detection limits than those observed with plasmid-based standards. Consequently, relying solely on plasmid standards may underestimate the true concentration of RNA targets [48,49]. This difference reflects the inherent instability of RNA molecules and the multi-step enzymatic process required for their detection, rather than a deficiency of the assay itself. By incorporating both standard types, the present study provides a comprehensive evaluation of assay performance under both ideal and practical diagnostic conditions, ensuring reliability for both laboratory and field-based epidemiological applications. Interestingly, a slight deviation from linearity was observed at the highest concentrations of the RNA transcript standard. This behavior is commonly reported in one-step RT-qPCR assays and is generally attributed to the additional reverse transcription step, RNA instability, and minor enzymatic saturation effects at high template input levels [49,50]. Importantly, this deviation was limited in magnitude and did not affect assay linearity, amplification efficiency, or quantitative reliability within the diagnostically relevant concentration range. For samples containing exceptionally high viral RNA loads, accurate quantification can be readily achieved through simple template dilution.

Field application further demonstrated the assay’s practical value for epidemiological surveillance and pathogen distribution analysis. A total of 257 golden pompano samples were collected from multiple coastal aquaculture sites in Guangdong and Guangxi provinces and tested for CAPRV RNA. These samples were simultaneously analyzed using the one-step TaqMan qPCR assay, which targets the N gene and conventional PCR developed in this study, and two-step TaqMan qPCR assay, which targets the N gene by Huang et al. [21], as well as the previously reported one-step TaqMan qPCR assay by Sun et al. (2025b) [9], which targets the G gene. The results showed that the N gene–based one-step TaqMan qPCR achieved a positive detection rate of 53.70% (138/257) and the N gene–based two-step TaqMan qPCR achieved a positive detection rate of 48.25% (124/257), whereas the G gene–targeting assay of Sun et al. (2025) [9] detected 45.14% (116/257), and conventional PCR detected only 34.63% (89/257). These results clearly demonstrate that the analytical sensitivity of one-step TaqMan qPCR assays is superior to that of conventional PCR. At the same time, the N gene–targeting one-step TaqMan qPCR assay developed in this study showed a higher positive detection rate compared with the G gene–targeting assay reported by Sun et al. [9], highlighting the stronger accuracy and sensitivity of the assay developed in this study. Importantly, the application of this assay across multiple farms and regions enabled a comparative assessment of CAPRV prevalence, providing valuable epidemiological insights into pathogen distribution in southern China’s marine aquaculture systems. Moreover, the differences in CAPRV prevalence across farms may reflect variations in management practices, environmental conditions, and fish health status. These differences suggest that certain farms may act as virus reservoirs, facilitating ongoing transmission, while others may be at early stages of infection, highlighting the importance of targeted biosecurity and monitoring to control viral spread in golden pompano aquaculture. Overall, CAPRV RNA was detected in samples from several locations, with the total positive rate exceeding that of the previous year, suggesting that the virus has likely become endemic in certain mariculture zones. Furthermore, viral RNA detection in clinically healthy fish indicates potential asymptomatic or carrier states, implying that subclinical infections may act as silent reservoirs facilitating viral persistence and transmission within farms—a phenomenon similarly reported for other fish rhabdoviruses [51,52]. These findings underscore the necessity of incorporating highly sensitive molecular diagnostic tools into routine biosecurity and health monitoring programs. Early detection of infected or carrier fish enables timely epidemiological intervention and informed management decisions, including stock segregation, water disinfection, and movement control, thereby mitigating further spread and reducing the economic impact of CAPRV outbreaks in golden pompano aquaculture.

In summary, the one-step TaqMan qPCR assay developed in this study provides a rapid, reliable, and high-sensitivity platform for the detection of CAPRV. By integrating improved molecular design, dual-standard validation, and field-based optimization, this method establishes a new benchmark for rhabdovirus diagnostics in aquaculture. Its application will facilitate routine epidemiological surveillance, comparative analysis of pathogen distribution across farms and regions, and early warning of emerging CAPRV outbreaks in southern China, thereby contributing to more effective disease management strategies and the sustainable development of marine fish farming systems.

## Figures and Tables

**Table 1:** Detail of probe and primers used in this study.

Primers and Probe	Sequence
Nke-F	5′-ATGTCTATGAGAGATACATTTGAGGG-3′
Nke-R	5′-CTAATCAAAGTAGCTGGAAAAGTCT-3′
CAPRV-N-F	5′-CCGGAATGACCATGATCGGA-3′
CAPRV-N-R	5′-CTTGGCAGATTCCACCAAAGA-3′
CAPRV-N-Probe	5′-FAM-TGCTCCTAGACTCTTTGCTGCCTGAGT-3′ BHQ1
N-F	5′-AGGAGTCCCAGAACTCGCA-3′
T7-N-R	5′-TAATACGACTCACTATAGGGATCAGCAAGAAGCTGGAAGG-3′

**Table 2:** The reproducibility of the CAPRV one-step TaqMan qPCR assay was evaluated using standard plasmid dilutions ranging from 2 × 10^0^ to 2 × 10^9^ copies/μL.

Plasmid Concentration (Copies/μL)	Intra-Assay Repeatability		Inter-Assay Repeatability	
	Ct Mean ( $\bar{x}$ ± SD)	Coefficient of Variation (CV%)	Ct Mean ( $\bar{x}$ ± SD)	Coefficient of Variation (CV%)
2 × 10^0^	34.86 ± 0.19	0.53	34.95 ± 0.34	0.96
2 × 10^1^	33.44 ± 0.56	1.66	33.38 ± 0.52	1.56
2 × 10^2^	29.87 ± 0.15	0.49	29.91 ± 0.30	1.01
2 × 10^3^	25.53 ± 0.06	0.24	25.58 ± 0.18	0.70
2 × 10^4^	22.15 ± 0.04	0.16	22.12 ± 0.13	0.58
2 × 10^5^	18.65 ± 0.07	0.35	18.71 ± 0.16	0.86
2 × 10^6^	15.19 ± 0.19	1.24	15.25 ± 0.24	1.43
2 × 10^7^	11.71 ± 0.11	0.91	11.68 ± 0.14	1.22
2 × 10^8^	8.19 ± 0.05	0.60	8.28 ± 0.09	1.06

SD: standard deviation.

**Table 3:** The reproducibility of the CAPRV one-step TaqMan qPCR assay was evaluated using in vitro-transcribed RNA standards ranging from 2 × 10^1^ to 2 × 10^9^ copies/μL.

In Vitro Transcription RNA Concentration (Copies/μL)	Intra-Assay Repeatability		Inter-Assay Repeatability	
	Ct Mean ( $\bar{x}$ ± SD)	Coefficient of Variation (CV%)	Ct Mean ( $\bar{x}$ ± SD)	Coefficient of Variation (CV%)
2 × 10^1^	39.52 ± 0.08	0.19	39.48 ± 0.25	0.63
2 × 10^2^	37.20 ± 0.24	0.64	37.32 ± 0.45	1.21
2 × 10^3^	33.79 ± 0.66	1.94	33.85 ± 0.72	1.67
2 × 10^4^	31.99 ± 0.30	0.94	32.12 ± 0.42	1.31
2 × 10^5^	28.31 ± 0.12	0.44	28.25 ± 0.28	0.99
2 × 10^6^	25.29 ± 0.23	0.89	25.41 ± 0.35	1.38
2 × 10^7^	20.89 ± 0.16	0.76	20.96 ± 0.18	0.86
2 × 10^8^	17.34 ± 0.13	0.72	17.42 ± 0.12	0.69

SD: standard deviation.

**Table 4:** Ct Value Comparison of Ten CAPRV Strains Detected by One-Step and Two-Step TaqMan N-Gene qPCR.

CAPRV Virus Strain	One-Step TaqMan N qPCR (Mean ± SD)	Two-Step TaqMan N qPCR (Mean ± SD)	ΔCt (Two-Step–One-Step)
2023	11.49 ± 0.27	13.87 ± 0.18	2.38
2024-ZJNS	20.48 ± 0.34	26.34 ± 0.27	5.86
2024-ZJTC	13.73 ± 0.11	17.04 ± 0.16	3.31
2024-GXBH	17.85 ± 0.21	27.83 ± 0.26	9.98
2025-ZJCT	15.35 ± 0.17	23.47 ± 0.18	8.12
2025-ZJDL	22.59 ± 0.14	28.83 ± 0.33	6.24
2025-ZHTM	18.32 ± 0.27	23.80 ± 0.28	5.48
2025-ZJLS	15.46 ± 0.23	18.30 ± 0.27	2.84
2025-GXFCG	16.18 ± 0.18	25.80 ± 0.28	9.62
2025-YJ	21.05 ± 0.18	29.32 ± 0.27	8.27

**Table 5:** Positive Detection Rates of CAPRV in Clinical Samples Collected from 2024 to 2025 Using Different Assays.

Methods	Positive Samples	Positive Rate (%)
One-step TaqMan N qPCR	138/257	53.70
Conventional N PCR	89/257	34.63
Two-step TaqMan N qPCR	124/257	48.25
One-Step TaqMan G qPCR	116/257	45.14

## Data Availability

The original contributions presented in this study are included in the article. Further inquiries can be directed to the corresponding author.
